# Age-Dependent Association of *TNFSF15*/*TNFSF8* Variants and Leprosy Type 1 Reaction

**DOI:** 10.3389/fimmu.2017.00155

**Published:** 2017-02-14

**Authors:** Vinicius M. Fava, Carolinne Sales-Marques, Alexandre Alcaïs, Milton O. Moraes, Erwin Schurr

**Affiliations:** ^1^Program in Infectious Diseases and Immunity in Global Health, Research Institute of the McGill University Health Centre, Montreal, QC, Canada; ^2^The McGill International TB Centre, Department of Human Genetics, McGill University, Montreal, QC, Canada; ^3^Laboratório de Hanseníase, Instituto Oswaldo Cruz, FIOCRUZ, Rio de Janeiro, Brazil; ^4^Laboratory of Human Genetics of Infectious Diseases, Necker Branch, Institut National de la Santé et de la Recherche Médicale U1163, Paris, France; ^5^Imagine Institute, University Paris Descartes, Paris, France; ^6^Giles Laboratory of Human Genetics of Infectious Diseases, Rockefeller Branch, Rockefeller University, New York, NY, USA; ^7^Department of Medicine, McGill University, Montreal, QC, Canada

**Keywords:** age at disease diagnosis, leprosy, excessive inflammatory response, *TNFSF8*, *TNFSF15*, type 1 reaction, association studies, Crohn’s disease

## Abstract

A current major challenge in leprosy control is the prevention of permanent disabilities. Host pathological inflammatory responses termed type 1 reaction (T1R) are a leading cause of nerve damage for leprosy patients. The environmental or inherited factors that predispose leprosy cases to undergo T1R are not known. However, studies have shown an important contribution of host genetics for susceptibility to T1R. We have previously identified variants encompassing the *TNFSF15*/*TNFSF8* genes as T1R risk factors in a Vietnamese sample and replicated this association in a Brazilian sample. However, we failed to validate in Brazilian patients the strong association of *TNFSF15*/*TNFSF8* markers rs6478108 and rs7863183 with T1R that we had observed in Vietnamese patients. Here, we investigated if the lack of validation of these variants was due to age-dependent effects on association using four independent population samples, two from Brazil and two from Vietnam. In the combined analysis across the four samples, we observed a strong association of the *TNFSF15*/*TNFSF8* variants rs6478108, rs7863183, and rs3181348 with T1R (*p*_combined_ = 1.5E−05, *p*_combined_ = 1.8E−05, and *p*_combined_ = 6.5E−06, respectively). However, the association of rs6478108 with T1R was more pronounced in leprosy cases under 30 years of age compared to the global sample [odds ratio (OR) = 1.95, 95% confidence interval (CI) = 1.54–2.46, *p*_combined_ = 2.5E−08 versus OR = 1.46, 95% CI = 1.23–1.73, *p*_combined_ = 1.5E−05]. A multivariable analysis indicated that the association of rs6478108 with T1R was independent of either rs7863183 or rs3181348. These three variants are known regulators of the *TNFSF8* gene transcription level in multiple tissues. The age dependency of association of rs6478108 and T1R suggests that the genetic control of gene expression varies across the human life span.

## Introduction

Leprosy is a chronic dermato-neurological infectious disease caused by *Mycobacterium leprae*. Due to leprosy control measures, the prevalence of the disease has been reduced to less than 1/10,000 of the global population. However, leprosy persists as major public health problem in sub-national foci. The incidence of new cases has shown only a gradual decline over the last 10 years and as of 2015 stood at 210,758 (1). A major goal of the global leprosy strategy is the reduction of disabilities due to leprosy. In leprosy, a major cause of tissue damage and permanent disability are host pathological inflammatory episodes termed type 1 reactions (T1Rs). Hence, translational research activities are dedicated toward a better understanding of T1Rs and their relationship with overall leprosy pathogenesis. The main dermato-pathological findings in T1R are an increased infiltrate of lymphocytes in the dermis, intragranuloma edema, and loss of the normal granuloma structure (2, 3). T1Rs are common and affect up to 50% of leprosy patients depending on the endemic settings with many patients presenting recurring episodes (4). The trigger for a T1R episode is not known, but studies have provided evidence of a genetic component in T1R susceptibility (4–8).

We have recently identified single-nucleotide variants (SNVs) in the vicinity of the neighboring genes *TNFSF15* and *TNFSF8* as risk factors of T1R (8). A set of SNVs associated with T1R in two Vietnamese and Brazilian samples correlated with *TNFSF8* gene expression levels (8). The T1R risk locus in Brazilians was restricted to SNVs physically encompassing the *TNFSF8* gene. However, the strongest association with T1R in the Vietnamese population was observed for two SNVs (rs6478108 and rs7863183) located distal to the *TNFSF8* gene. Although divergences in the linkage disequilibrium pattern across populations may account for the differences of T1R association with SNVs in the *TNFSF8* gene region, age at onset of disease was not taken into account. It was shown by twin studies that the heritability of major immune traits is strongly age dependent (9). Likewise, we have previously shown for the *LTA* and *PARK2* genes that the age at leprosy diagnosis is crucial for the association of certain SNVs and leprosy *per se* (10, 11). The age at leprosy diagnosis for T1R cases in the Brazilian sample was two times higher than the age at leprosy diagnosis in the Vietnamese sample. Therefore, the lack of validation for T1R and *TNFSF8* distal SNVs in the Brazilian sample may reflect age-dependent mechanisms of gene expression. To address the age dependency of genetic risk factors in T1R, we revisited the association of the *TNFSF15*/*TNFSF8* locus with T1R by focusing on three key T1R risk SNVs (rs3181348, rs6478108, and rs7863183) in the Brazilian and Vietnamese samples and evaluated the age-dependent strength of the evidence for association.

## Materials and Methods

### Ethics Approval Statement

Written informed consent was obtained from all subjects participating in the study. All minors assented to the study, and a parent or guardian provided the informed consent on their behalf. The study was approved by ethics committees of the participating centers and conducted according to the principles expressed in the Declaration of Helsinki.

### Population Sample

For the current study, we evaluated four independent populations. The Vietnam I and the Brazil I population samples have been described previously (8). Briefly, the Vietnam I sample consisted of 224 T1R-affected offspring from our family-based design approach (8). The Brazil I sample comprised 758 leprosy cases enrolled from the Central-West Region of Brazil with 374 of these being T1R affected and the remaining 384 being T1R free (8). To study the effect of age at leprosy diagnosis on the strength of association of T1R with the *TNFSF15*/*TNFSF8* locus, two new population samples were enrolled and named Vietnam II and Brazil II (Table 1) (12). The Vietnam II population sample comprised 816 leprosy-affected patients of which 253 presented T1R, while the remaining 563 were T1R free. The Brazil II population sample comprised 136 T1R-affected and 170 T1R-free leprosy patients recruited from Rio de Janeiro in the Southeast region of Brazil. As T1R affects predominantly leprosy patients classified as borderline within the Ridley and Jopling leprosy spectrum, T1R-free controls were also selected mainly from the borderline form of the disease. Details regarding the clinical characteristics of the Vietnam II and Brazil II samples can be found in Table S1 in Supplementary Material.

**Table 1:** **Age at leprosy diagnosis of four studied samples**.

	T1R-affected			T1R-free		
	Age at leprosy diagnosis (years)			Age at leprosy diagnosis (years)		
	Patients	Median	Mean (SD)	Patients	Median	Mean (SD)
**Discovery**						
Vietnam I^a^	224	18	19.7 (7.2)	n.a.	n.a.	n.a.
**Replication**						
Vietnam II	262	25	26.5 (10.1)	576	24	23.8 (7.8)
**Validation**						
Brazil I^a^	374	47	46.7 (15.8)	384	40	41.8 (16.9)
Brazil II	136	44	43.0 (17.8)	170	41	41.1 (17.6)

*T1R, type 1 reaction; n.a., not applicable*.

*^a^Clinical characteristics and univariable analysis for association with T1R were previously reported for these samples (8)*.

### Genotyping

For the current study, three SNVs previously associated with T1R (rs6478108, rs7863183, and rs3181348) were genotyped using the SEQUENOM MassARRAY platform or obtained by a larger scale effort using the high-throughput Illumina platform (13). The five SNVs presented call rate >0.9 and were in Hardy–Weinberg equilibrium with *p* values >0.05 in the control groups in all study phases.

### Statistical Approach

To investigate if *TNFSF15*/*TNFSF8* SNVs’ association with T1R was restricted to an early age at leprosy diagnosis, we performed a stratified analysis dividing the T1R-affected cases into three age categories: (i) children and young adults up to 29 years old, (ii) adults from 30 to 60 years old, and (iii) seniors above 60 years old. The association analysis was carried out in each age strata at first, and finally, combined analysis with the overall sample was applied for comparison. For the Vietnam I sample, pseudo-sib controls were generated based on our family-based approach data (8). Briefly, the non-transmitted allele from parents to T1R-affected offspring in a transmission disequilibrium test was used to create up to three unaffected pseudo-sibs per family, one for each possible genotype (14). The pseudo-sibs matched the clinical characteristics of the offspring of which it derived from; therefore, it can only be compared in a matched case–control design. This strategy was used to estimate SNVs’ risk in odds ratio (OR) format and their respective confidence intervals (CIs) using conditional logistic regression in SAS software (version 9.3; SAS Institute). For the replication and validation phases, both additive and dominant models of inheritance were tested using the same reference allele for each SNV in PLINK 1.07 and the best *p*-value was displayed (15). In each age at leprosy diagnosis category, T1R-affected cases were compared to the totality of T1R-free cases as controls. No adjustments were made in the logistic procedures as gender was not associated with T1R risk and clinical form of leprosy was a criterion for sample selection. The two-sided test is given for all samples as the research question focused on the effect of an allele independent of the direction, which for the Brazilian sample was different in the initial T1R versus *TNFSF8*/*TNFSF15* study (8).

To perform a combined analysis in each age category and the overall sample, we performed a meta-analysis in the software METAL (16). The contribution of each sample to the final statistic was weighted according to the effect size (β) derived from the logistic procedure and its respective SEs. To estimate population heterogeneity, the *I*^2^ statistic was calculated. The *I*^2^ values correspond to the percentage of variance in a meta-analysis that is attributed to study heterogeneity. Minor allele frequencies, Hardy–Weinberg equilibrium, and linkage disequilibrium structure were calculated in Haploview 4.2 (17).

The multivariable analysis was carried out with stepwise conditional logistic regression in SAS software. The haplotype analysis was performed with the THESIAS software (18). Briefly, T1R-affected subjects were compared to T1R-free (Vietnam II, Brazil I, and Brazil II) or with the untransmitted haplotypes from parents to offspring (Vietnam I) in a matched case–control design.

## Results

**Figure 1:**
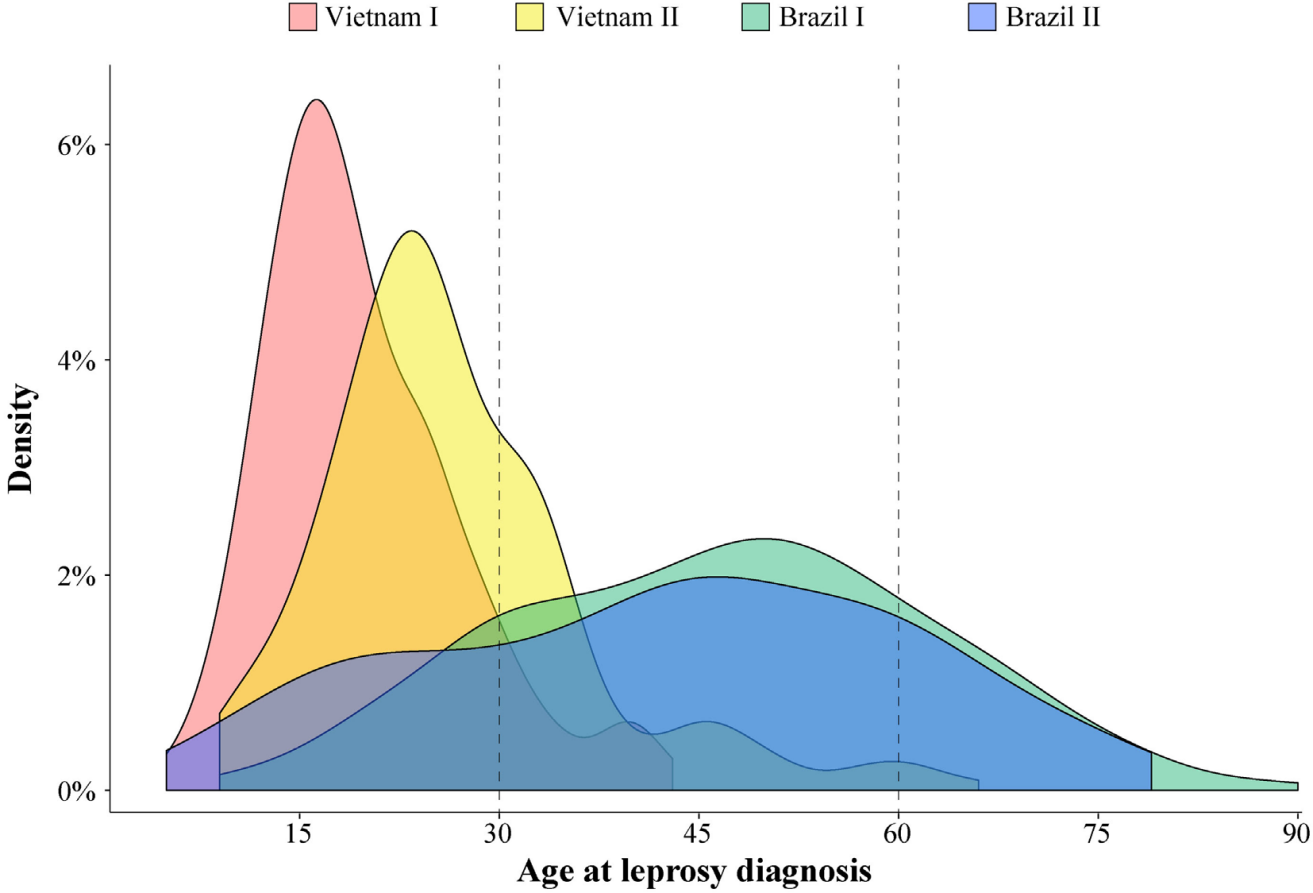
**Age at leprosy diagnosis density in leprosy type-1 reaction (T1R)-affected patients**. The age distribution of 996 T1R-affected leprosy cases from the 4 studied populations is plotted according to the density of cases.

A substantial variation of age at leprosy diagnosis for T1R-affected cases was observed between the Vietnamese and Brazilian samples (Figure 1). The largest difference in age at leprosy diagnosis was observed for the Vietnam I and the Brazil I samples (median of 18 and 47 years, respectively; *p* < 0.0001), while the difference between the two Brazilian samples (Brazil I and Brazil II) was small (median of 47 and 44 years, respectively; *p* = 0.03). In addition, for the Brazilian patients, the age at diagnosis was spread over a larger age range (Figure 1). By contrast, the Vietnamese samples presented a narrower age-at-diagnosis distribution with the majority of T1R-affected cases being diagnosed before 30 years of age (Figure 1).

### The Association of rs6478108 with T1R Is Age Dependent

**Figure 2:**
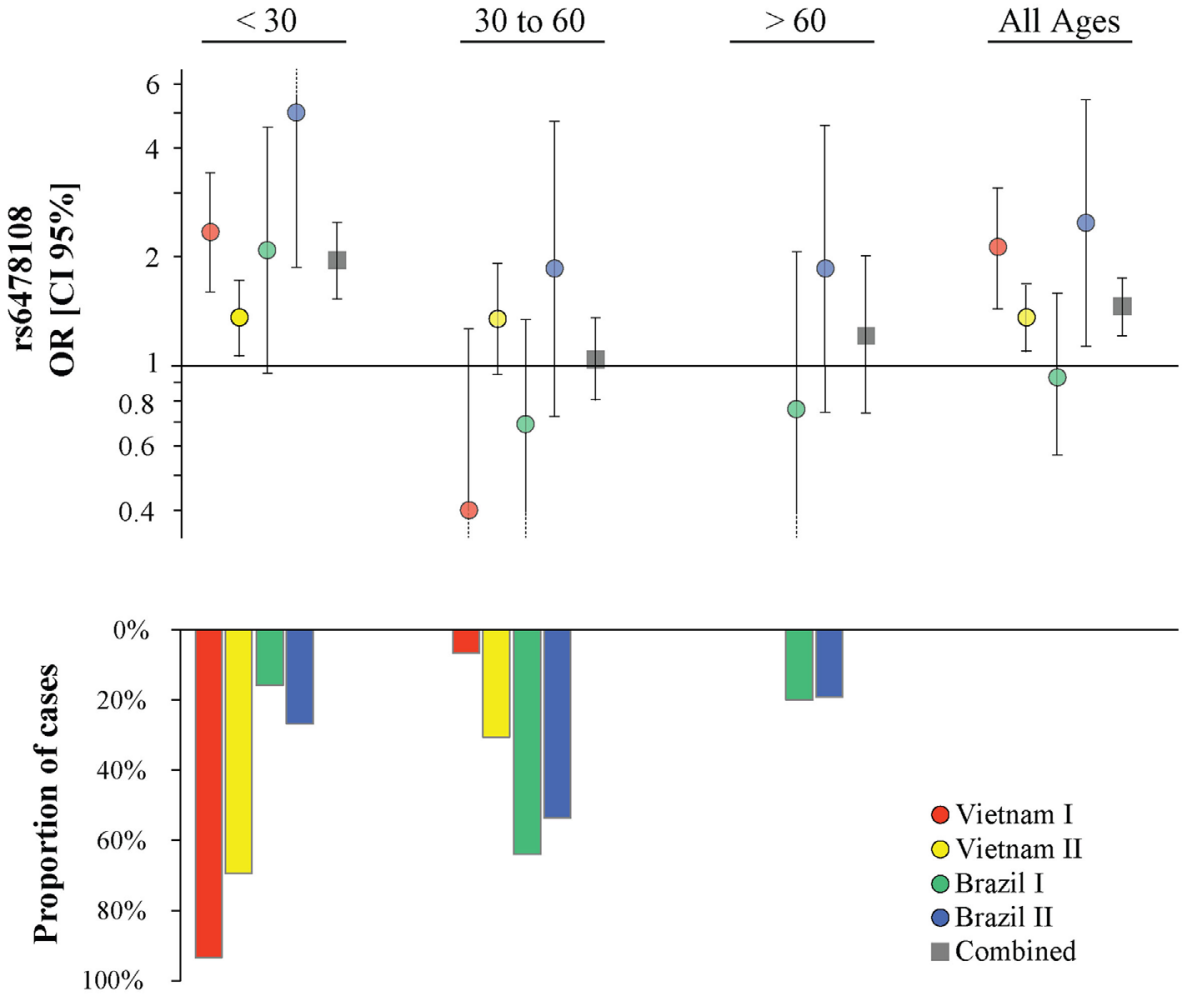
**Age at leprosy diagnosis stratified association analysis of rs6478108 in *TNFSF15* and type-1 reaction (T1R)**. The top panels indicate the strength of genetic effect as odds ratio (OR) for the four populations and the combined sample. Results are shown for three age strata and the entire sample regardless of age. Each colored circle represents the OR for each population sample with vertical lines delimiting their respective 95% confidence intervals (CIs). Dotted lines in the CI represent values that extend beyond the *y*-axis limits. The bottom panel presents the percentage of T1R-affected cases in each age stratum.

The G-allele of the *TNFSF15* intron variant rs6478108 had previously been associated with T1R in the Vietnam I population. However, we failed to validate rs6478108 as a T1R risk factor in the Brazil I sample, despite good power to detect an effect of similar size as found in Vietnam I. Indeed, given the observed minor allele frequency of 0.31 of Brazil I and assuming a type 1 error of 0.05, power was 95.7% (additive model) and 81.5% (dominant model) to detect the lower boundary risk (OR = 1.45) observed for Vietnam I (Table 2; Figure 2). By contrast, the G-allele of rs6478108 was significantly associated with T1R risk in the Vietnam II (*p* = 0.007) and Brazil II samples (*p* = 0.02) even though these populations, compared to Brazil I, presented a lower number of T1R-affected subjects (Figure 2; Table 2).

**Table 2:** **Effect of age at leprosy diagnosis on association of rs6478108 with T1R**.

		rs6478108-G allele	
Age at leprosy diagnosis	Samples	OR (95% CI)	*p* Value
<30	Vietnam I	2.34 (1.59–3.45)	1.50E−05
	Vietnam II	1.37 (1.06–1.75)	0.02
	Brazil I	2.08 (0.96–4.50)	0.06
	Brazil II	4.97 (1.88–13.2)	0.001

	Combined	1.95 (1.54–2.46)	2.50E−08
	*I*^2^ (*p* value)	55.3	0.06

30–60	Vietnam I	0.40 (0.13–1.28)	0.12
	Vietnam II	1.35 (0.94–1.94)	0.1
	Brazil I	0.69 (0.36–1.33)	0.27
	Brazil II	1.85 (0.73–4.66)	0.19

	Combined	1.04 (0.81–1.34)	0.75
	*I*^2^ (*p* value)	53.6	0.07

>60	Vietnam I	n.a.	n.a.
	Vietnam II	n.a.	n.a.
	Brazil I	0.76 (0.29–2.05)	0.59
	Brazil II	1.85 (0.75–4.57)	0.18

	Combined	1.21 (0.74–2.00)	0.44
	*I*^2^ (*p* value)	0	0.43

All ages	Vietnam I	2.13 (1.45–3.13)	2.10E−04
	Vietnam II	1.36 (1.09–1.70)	0.007
	Brazil I	0.93 (054–1.57)	0.77
	Brazil II	2.48 (1.14–5.37)	0.02

	Combined	1.46 (1.23–1.73)	1.50E−05
	*I*^2^ (*p* value)	54.9	0.06

*OR, odds ratio; CI, confidence interval; n.a., not applicable; T1R, type-1 reaction*.

The proportion of T1R-affected cases diagnosed with leprosy before 30 years for Vietnam I and Vietnam II was approximately 93 and 70%, respectively, while in Brazil I and Brazil II, this age category represented only 16 and 27%, respectively, of T1R patients. We hypothesized that the small proportion of patients younger than 30 years at leprosy diagnosis in the Brazil I sample could be masking a true association of rs6478108 and T1R. When a stratified analysis by age at leprosy diagnosis, the under 30-year at-diagnosis category displayed the most significant evidence for association of rs6478108 with T1R across all samples. Consequently, in the combined analysis of all samples, the under 30-year at-diagnosis subset presented a stronger OR and a more significant *p*-value than the overall combined sample (OR = 1.95, *p* = 2.5 × 10^−8^ and OR = 1.46, *p* = 1.5 × 10^−5^, respectively). The most striking impact of age at leprosy diagnosis was observed for the Brazilian I sample (Table 2; Figure 2). In Brazil I, rs6478108 was not associated with T1R in the overall analysis with the G-allele tending toward protection for T1R (OR = 0.93, *p* = 0.77). However, in the under 30-year age group, the G-allele of rs6478108 showed a strong trend toward association with T1R (OR = 2.08, *p* = 0.06; Table 2; Figure 2). The effect of age was also strong in the Brazil II sample where the risk was twofold higher in the under 30-year at-diagnosis group compared to the overall sample (OR = 4.97, *p* = 0.001 and OR = 2.48, *p* = 0.02, respectively). Although Brazil I and Brazil II were analyzed separately, linkage disequilibrium analysis of the Brazil II revealed near identity with the Brazilian I sample (Figure S1 in Supplementary Material). A combined analysis of both Brazilian samples confirmed the age-dependent association of rs6478108 with T1R (Figure S2 in Supplementary Material). No significant association of rs6478108 with T1R was observed for the age groups of 30–60 years and over 60 years at leprosy diagnosis (Table 2; Figure 2).

In contrast to rs6478108, both rs7863183 and rs3181348 did not present a strong age-at-diagnosis effect of their association with T1R (Table S2 and Figures S3 and S4 in Supplementary Material). Indeed, rs7863183 and rs3181348 remained significantly associated with T1R in the age-at-diagnosis category from 30 to 60 years (*p* = 0.04 for both), suggesting a difference in the underlying mechanism of T1R susceptibility mediated by rs6478108 and rs7863183 (Table S2 and Figures S3 and S4 in Supplementary Material). To follow-up on this observation, we conducted a multivariable analysis to investigate independence of association of the three SNVs with T1R.

### Two Independent Associations with T1R in the *TNFSF8*/*TNFSF15* Locus

When the three SNVs were included in a multivariable model, rs6478108 and rs7863183 maintained the significant association with T1R, while rs3181348 lost significance (Table 3). The association of rs6478108 and rs7863183 with T1R was also observed in the <30 years group at leprosy diagnosis reinforcing the observation that the signal captured by these two variants is independent (Table 3). The SNV selected first by the multivariable procedure flipped when only the <30 years group was evaluated. In the overall sample, rs7863183 was the most significant SNV, while rs6478108 was selected first in the <30 years group. This observation is in agreement with the stronger effect of rs6478108 in early-onset cases of T1R. As the multivariable analysis suggested that the association of rs6478108 and rs7863183 with T1R was independent, we performed a haplotype analysis to evaluate the combined effect of these two variants on T1R susceptibility. We observed that the (G–T) haplotype, i.e., absence of T1R susceptibility alleles (A–C) for rs6478108 and rs7863183, was significantly protective for T1R in the overall sample (*p* = 0.0009; Table 4). However, the most striking observation was in the <30 years group at leprosy diagnosis category where all the alternative haplotypes (A–C, A–T, and G–C) were protective when compared to the haplotype containing both T1R risk alleles (G–T) for rs6478108 and rs7863183 (Table 4).

**Table 3:** **Multivariable analysis in the combined population samples**.

		All ages			<30		
Single-nucleotide variant	Allele	*p* Value_univariable_	Odds ratio (OR) [95% confidence interval (CI)]	*p* Value_multivariable_	*p* Value_univariable_	OR (95% CI)	*p* Value_multivariable_
rs6478108	G	1.50E−05	1.46 (1.23–1.73)	0.02	2.50E−08	1.95 (1.54–2.46)	0.0007
rs7863183	T	1.80E−05	1.35 (1.18–1.56)	0.004	1.10E−04	1.47 (1.21–1.79)	0.01
rs3181348	G	6.50E−06	1.40 (1.21–1.62)	0.28	5.80E−06	1.63 (1.32–2.01)	0.72

**Table 4:** **Haplotype analysis of rs6478108 and rs7863183**.

	All ages		<30	
rs6478108–rs7863183	Odds ratio (OR) [95% confidence interval (CI)]	*p* Value	OR (95% CI)	*p* Value
G–T	Baseline		Baseline	
G–C	0.76 (0.55–1.06)	0.10	0.70 (0.55–0.89)	0.004
A–T	0.75 (0.61–0.92)	0.005	0.80 (0.68–0.93)	0.005
A–C	0.73 (0.61–0.88)	0.0009	0.83 (0.74–0.94)	0.004

## Discussion

Here, we showed the age-dependent association of rs6478108 with T1R in ethnically distinct and geographically separated populations. By contrast, the association of rs3181348 and rs7863183 with T1R was not restricted to early-onset leprosy cases. Albeit the model selected first by the multivariable analysis indicated rs6478108 and rs7863183 as independent signals of association with T1R, this model was not statistically different from the model encompassing rs6478108 and rs3181348. This suggests that the association of rs6478108 with T1R was independent of both rs7863183 and of our previous findings reporting the association of rs3181348 with T1R (8). While rs6478108 is one of the two independent signals of association with T1R, the second signal could not be unambiguously assigned to rs7863183 or rs3181348 as both captured the same information. The estimated risk effect for both rs6478108 and rs7863183 in the overall population (OR = 1.46 and OR = 1.35, respectively) presented borderline significant *p* values for heterogeneity across the samples in the *I*^2^ test (Table 2; Table S2 in Supplementary Material). This observation may be due to winner’s curse where the risk effects estimated in an initial scan are stronger than the risk effect observed in replication samples (19, 20). Irrespectively, the consistent association of *TNFSF8*/*TNFSF15* variants with T1R in multiple populations reinforces the finding of rs7863183 and rs6478108 as global risk factor for T1R. The haplotype analysis indicated that subjects carrying T1R risk alleles in both rs6478108 and rs7863183 locus were significantly more susceptible to T1R compared to subjects carrying a T1R risk allele only in one of the two loci. The two SNVs independently associated with T1R have been reported as expression quantitative trait loci. Both rs6478108 and rs7863183 are associated with *TNFSF8* gene expression levels in peripheral blood (21) and in circulating monocytes (22) where the T1R risk alleles rs6478108-G and rs7863183-T correlated with higher gene expression. Interestingly, rs6478108 was also associated with *TNFSF15* gene expression in a tissue dependent manner (23). While the rs6478108-G allele correlated with higher *TNFSF15* expression in the esophagus and the lungs, the rs6478108-A allele correlated with higher *TNFSF15* expression in whole blood and in the liver (23).

The rs6478108 was originally reported as a leprosy *per se* susceptibility factor by a genome-wide association study in a Chinese population sample (24). Thereafter, subsequent studies failed to replicate the rs6478108 association with leprosy *per se* in independent populations (25, 26). Our group observed association of rs6478108 only with T1R but not leprosy *per se*, and here, we showed that the strength of association was strongly age dependent. The *TNFSF15*/*TNFSF8* region is a well-known locus associated with Crohn’s disease (CD) susceptibility (27). Multiple variants including rs6478108 and rs7863183 have been identified as risk factors for CD in distinct populations. The *TNFSF15*/*TNFSF8* locus was strongly implicated in CD in populations from East Asian origin where the T1R risk alleles rs6478108-G and rs7863183-T were shown to be strong CD risk factors (*p* = 2.97E−45; OR = 1.90, CI 95% = 1.73–2.07 and *p* = 2.30E−25; OR = 1.56, CI 95% = 1.44–1.70, respectively) (27). Interestingly, in a candidate gene approach, we recently showed that SNVs in the *LRRK2* gene presented the same risk alleles for T1R and CD (7, 28). Taken together, our results indicate a shared genetic component predisposing an individual to pathological inflammatory responses in leprosy and inflammatory bowel disease.

The mechanisms of genetic predisposition to disease are often closely connected with the age at onset of the disease. The interplay of age at onset and genetic control of disease is well known in neurodegenerative disorders. For instance, mutations in the *PARK2* gene are classic examples of early-onset Parkinson’s disease, while mutations in the *LRRK2* gene are the most frequent cause of late-onset autosomal-dominant Parkinson’s disease (29, 30). In infectious diseases, the effect of age on the genetic control of disease is further complicated by the necessity of exposure and infection by the causative pathogen. Length and intensity of exposure to the pathogen, as well as strain virulence, are factors that may affect early or late disease manifestation. With the advent of next-generation sequencing, studies have identified single-gene mutations as cause of extreme early-onset mycobacterial and/or viral infection diseases (31–33). While these mutations are rare and family specific, the cumulative effect of rare variants on early-onset infectious disease remains unknown. It is possible that early-onset infectious disease is controlled by multiple rare and common variants in several genes that together have moderate penetrance. The genetic control for early-onset infectious disease is distinct from late-onset/idiopathic infections (34). In late-onset cases of infection, a long history of host–environment interactions, the epigenome, somatic mutations, and cell senescence are more likely to contribute to host genetic susceptibility (35).

In conclusion, we have shown that a group of SNVs that regulate *TNFSF15* and *TNFSF8* gene expression are associated with T1R in four independent populations. While the association tagged by rs7863183 and rs3181348 was observed in all age categories, the rs6478108 association with T1R was dependent on the age at leprosy diagnosis. The age dependency of association suggests that the genetic control of gene expression varies across the human life span.

## Author Contributions

VF, AA, and ES designed the study; VF performed the data analysis; VF, ES, CS-M, and MM interpreted the results; VF and ES wrote the manuscript; CS-M and MM reviewed the manuscript.

## Conflict of Interest Statement

The research was conducted in the absence of any commercial or financial relationships that could be construed as a potential conflict of interest.
