# Supplementary figures and images for: Age-Dependent Association of TNFSF15/TNFSF8 Variants and Leprosy Type 1 Reaction
Source: Front Immunol. 2017 Feb 14;8:155. doi: 10.3389/fimmu.2017.00155 (PMC5306391; doi:10.3389/fimmu.2017.00155)

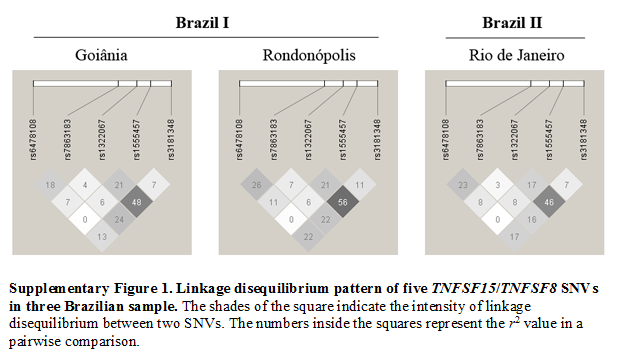

Supplement: Supplementary file 1 [file Image_1.TIF]

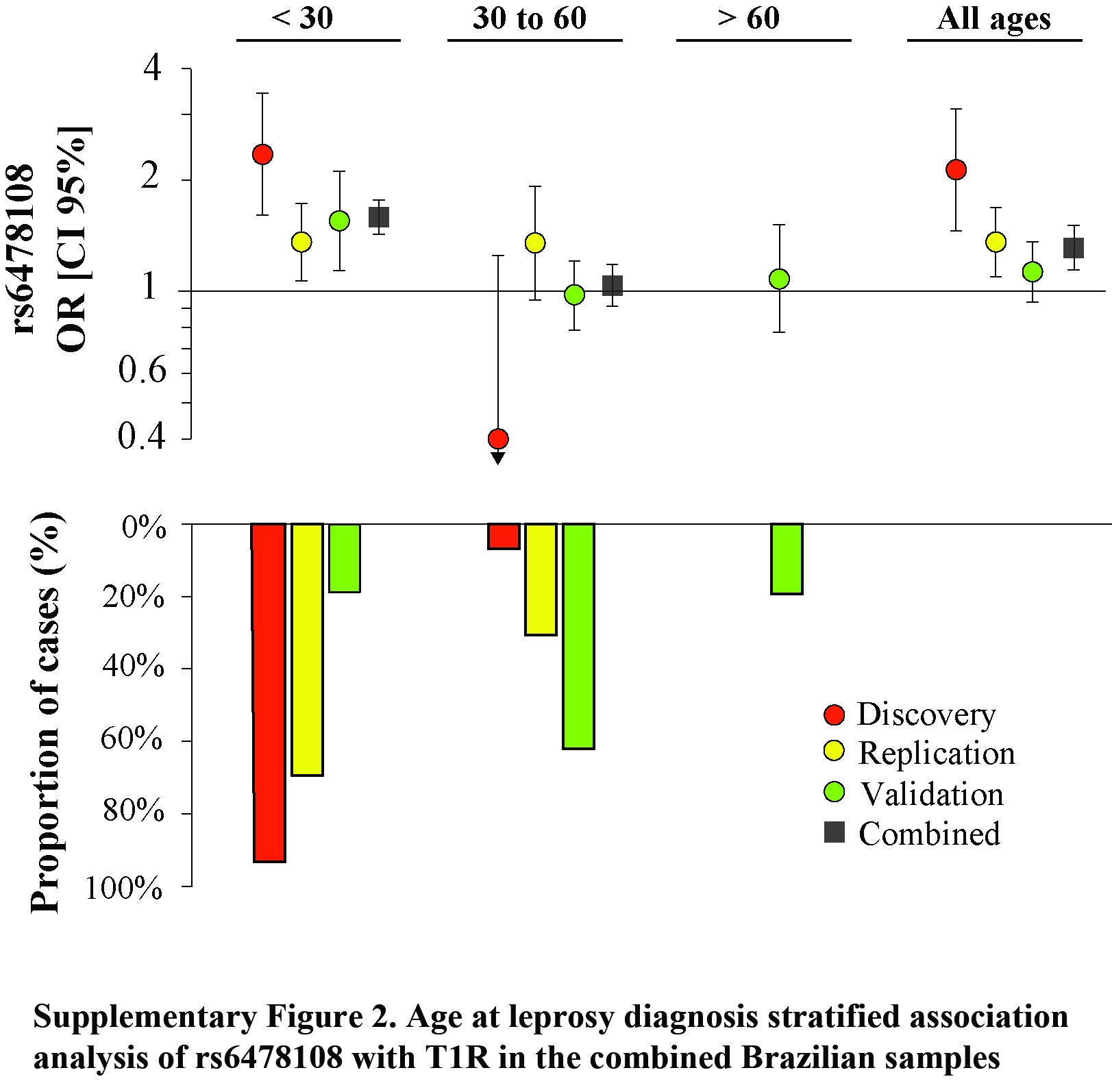

Supplement: Supplementary file 2 [file Image_2.TIF]

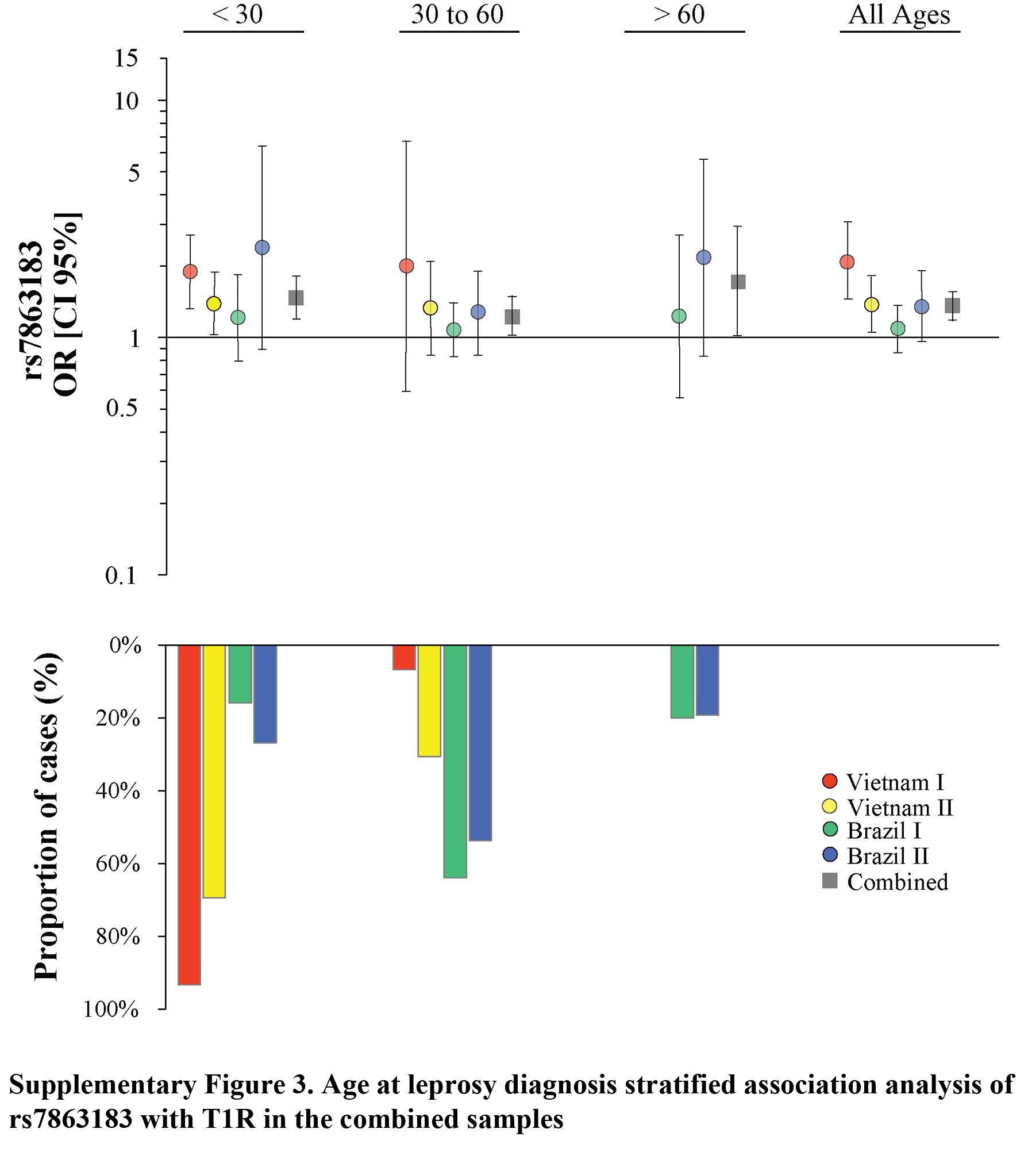

Supplement: Supplementary file 3 [file Image_3.TIF]

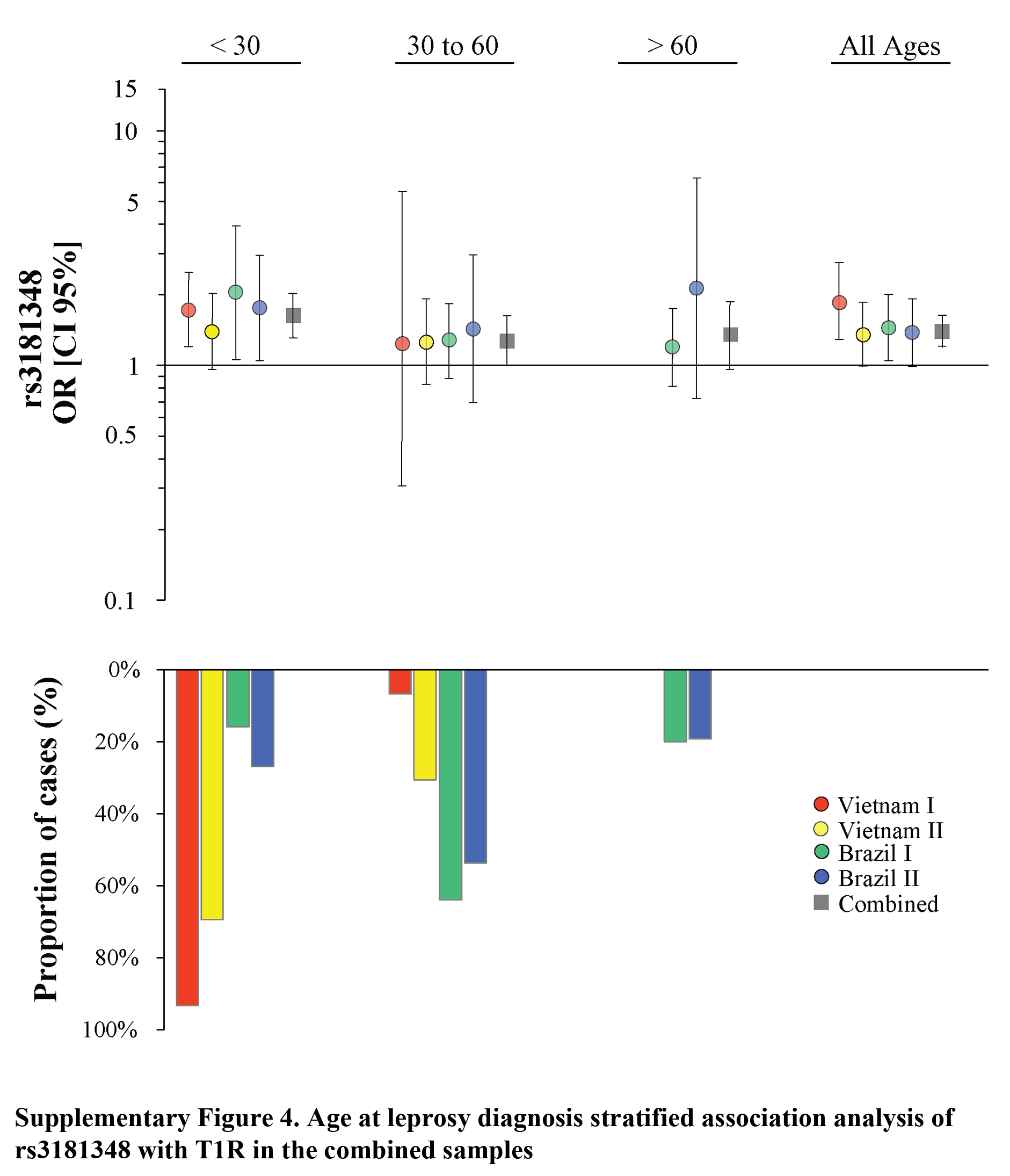

Supplement: Supplementary file 4 [file Image_4.TIF]
