# Supplementary material for: Age-Dependent Association of TNFSF15/TNFSF8 Variants and Leprosy Type 1 Reaction
Source: Front Immunol. 2017 Feb 14;8:155. doi: 10.3389/fimmu.2017.00155 (PMC5306391; doi:10.3389/fimmu.2017.00155)
Supplement: Supplementary file 5 [file Table_1.DOCX]

| **Supplementary Table 1. Clinical subtype of Vietnam II and Brazil II samples.** | | | | | | | |
| --- | --- | --- | --- | --- | --- | --- | --- |
|  |  |  | **Vietnam II** | |  | **Brazil II** | |
| Clinical Subtype *^a^* |  |  | T1R-Affected | T1R-free |  | T1R-Affected | T1R-free |
| TT |  |  | 2 (0.8) | 2 (0.4) |  | - | - |
| BT |  |  | 43 (17.0) | 197 (35.0) |  | 38 (27.9) | 94 (63.5) |
| BB |  |  | 99 (39.1) | 168 (29.8) |  | 37 (27.2) | 17 (11.5) |
| BL |  |  | 98 (38.7) | 185 (32.8) |  | 49 (36.0) | 25 (16.9) |
| LL |  |  | 9 (3.6) | 9 (1.6) |  | 8 (5.9) | 8 (5.4) |
| I |  |  | 2 (0.8) | 2 (0.4) |  | 4 (3.0) | 4 (2.7) |
| Total |  |  | 253 | 563 |  | 136 | 148 |
| Abbreviations: T1R, type-1 reaction; BB, borderline leprosy; BL, borderline lepromatous leprosy; BT, borderline tuberculoid leprosy; I, indeterminate; LL, lepromatous leprosy. | | | | | | | |
| *^a^* Clinical subtype of leprosy according to the Ridley and Jopling scale. | | | | | | | |
