# Supplementary material for: Age-Dependent Association of TNFSF15/TNFSF8 Variants and Leprosy Type 1 Reaction
Source: Front Immunol. 2017 Feb 14;8:155. doi: 10.3389/fimmu.2017.00155 (PMC5306391; doi:10.3389/fimmu.2017.00155)
Supplement: Supplementary file 6 [file Table_2.DOCX]

| **Supplementary table 2. Age at leprosy onset stratified association analysis ofrs7863183 and rs3181348 with T1R** | | | | | | |
| --- | --- | --- | --- | --- | --- | --- |
|  |  | **rs7863183-T allele** | |  | **rs3181348-G allele** | |
| Age at leprosy diagnosis | Samples | OR (95% CI) | *p* value |  | OR (95% CI) | *p* value |
| < 30 | Vietnam I | 1.89 (1.32 - 2.70) | 5.00E-04 |  | 1.72 (1.20 - 2.48) | 3.00E-03 |
|  | Vietnam II | 1.38 (1.02 - 1.88) | 0.04 |  | 1.39 (0.97 - 1.98) | 0.07 |
|  | Brazil I | 1.21 (0.80 - 1.83) | 0.37 |  | 2.05 (1.06 - 3.93) | 0.03 |
|  | Brazil II | 2.39 (0.90 - 6.34) | 0.08 |  | 1.76 (1.05 - 2.93) | 0.03 |
|  | Combined | 1.47 (1.21 - 1.79) | 1.10E-04 |  | 1.63 (1.32 - 2.01) | 5.80E-06 |
|  | I^2^ (*p* value) | 30.3 | 0.22 |  | 0 | 0.84 |
| 30 to 60 | Vietnam I | 2.00 (0.60 - 6.62) | 0.26 |  | 1.30 (0.31 - 5.41) | 0.72 |
|  | Vietnam II | 1.33 (0.85 - 2.07) | 0.21 |  | 1.26 (0.83 - 1.91) | 0.28 |
|  | Brazil I | 1.08 (0.84 - 1.39) | 0.56 |  | 1.27 (0.89 - 1.82) | 0.19 |
|  | Brazil II | 1.28 (0.86 - 1.90) | 0.22 |  | 1.43 (0.70 - 2.94) | 0.33 |
|  | Combined | 1.22 (1.01 - 1.47) | 0.04 |  | 1.27 (1.00 - 1.61) | 0.04 |
|  | I^2^ (*p* value) | 0 | 0.52 |  | 0 | 1 |
| > 60 | Vietnam I | n.a. | n.a. |  | n.a. | n.a. |
|  | Vietnam II | n.a. | n.a. |  | n.a. | n.a. |
|  | Brazil I | 1.23 (0.56 - 2.68) | 0.61 |  | 1.20 (0.82 - 1.74) | 0.35 |
|  | Brazil II | 2.17 (0.84 - 5.59) | 0.11 |  | 2.13 (0.73 - 6.23) | 0.17 |
|  | Combined | 1.71 (1.01- 2.89) | 0.04 |  | 1.34 (0.96 - 1.86) | 0.09 |
|  | I^2^ (*p* value) | 0 | 0.53 |  | 0 | 0.47 |
| All ages | Vietnam I | 2.08 (1.47 - 3.03) | 2.00E-04 |  | 1.85 (1.30 - 2.70) | 0.003 |
|  | Vietnam II | 1.38 (1.05 - 1.81) | 0.02 |  | 1.35 (1.00 - 1.85) | 0.05 |
|  | Brazil I | 1.09 (0.87 - 1.36) | 0.46 |  | 1.43 (1.04 - 1.97) | 0.03 |
|  | Brazil II | 1.37 (0.97 - 1.91) | 0.07 |  | 1.38 (0.99 - 1.91) | 0.06 |
|  | Combined | 1.35 (1.18 - 1.56) | 1.80E-05 |  | 1.40 (1.21 - 1.62) | 6.50E-06 |
|  | I^2^ (*p* value) | 60.3 | 0.04 |  | 1.4 | 0.40 |
| Abbreviations: OR, odds ratio; CI, confidence interval; n.a.., not applicable | | | | | | |
